# Arthroscopic Implantation of a Cell-Free Bilayer Scaffold for the Treatment of Knee Chondral Lesions: A 2-Year Prospective Study

**DOI:** 10.1177/19476035241232061

**Published:** 2024-03-19

**Authors:** Rimtautas Gudas, Mantas Staškūnas, Justinas Mačiulaitis, Emilė Gudaitė, Ieva Aleknaite-Dambrauskiene

**Affiliations:** 1 Department of Orthopedics and Traumatology, Hospital of Lithuanian University of Health Sciences, Kaunas Clinics, Kaunas, Lithuania; 2 Advanced Cell Therapy Unit, Physiology and Pharmacology Department, Lithuanian University of Health Sciences, Kaunas, Lithuania; 3 Institute of Cardiology, Lithuanian University of Health Sciences, Kaunas, Lithuania; 4 Lithuanian University of Health Sciences, Kaunas, Lithuania; 5 Institute of Mechatronics, Kaunas University of Technology, Kaunas, Lithuania

**Keywords:** articular cartilage, knee, cartilage regeneration, cartilage repair, cell-free bilayer scaffold

## Abstract

**Objective:**

The main objective of this study is to assess the safety and clinical efficacy of a cell-free bilayer scaffold (MaioRegen Chondro+ by Fin-Ceramica) in patients affected by chondral knee lesions of different origin and localization.

**Design:**

Thirty-one patients with focal chondral lesions of the knee were arthroscopically treated with MaioRegen Chondro+. All patients were prospectively evaluated for a minimum of 2 years using the International Knee Documentation Committee (IKDC) Questionnaire and the Tegner Activity Scale. Cartilage repair was assessed based on the Magnetic Resonance Observation of Cartilage Repair Tissue (MOCART) 2.0 score at 12 months. Follow-up at 36 months was available for 25 out of 31 patients.

**Results:**

From baseline to 6-, 12-, and 24-month follow-up, IKDC score significantly improved by 19.5 ± 7.27 (95% confidence interval [CI]: 16.9-22.2, *P* < 0.001), 30.8 ± 7.63 (95% CI: 28.0-33.6, *P* < 0.001), and 36.2 ± 8.00 points (95% CI: 33.3-39.2, *P* < 0.001), respectively. Tegner scores documented a substantial clinical improvement as early as 12 months after surgery (change of −0.6 ± 0.62; 95% CI: −0.8 to −0.4, *P* < 0.001), reaching the preinjury values. There was a statistically significant increase in the MOCART scores (*P* < 0.001). Comparable results were observed regardless of preintervention demographic characteristics, lesion site or etiology, or the number of treated sites. Notably, the significant clinical benefit was maintained in a subset of patients who reached 3-year follow-up. No adverse events were reported in the entire analyzed population.

**Conclusion:**

MaioRegen Chondro+ is a safe and effective device for the treatment of knee chondral lesions, enabling a significant clinical improvement for at least 2 years.

## Introduction

Articular cartilage repair is biologically challenging due to its highly complex, avascular, aneural and alymphatic nature, which greatly limits its regenerative potential.^1^ Traditional surgical approaches to address chondral injuries include bone marrow stimulation procedures (such as microfracture), aimed at stimulating the access to the cartilage lesion of mesenchymal stem cells from the medullary cavity of the subchondral bone,^2,3^ and procedures promoting cartilage formation, such as autologous chondrocyte implantation. If the entire osteochondral unit needs to be restored, osteochondral autograft or allograft transplantation can be performed.^4,5^ Despite an increase in research focus on the treatment techniques available, no consensus has been established about the best option.^6^

In the past 20 years, regenerative scaffold-based procedures have emerged as a potential therapeutic option for the treatment of chondral and osteochondral defects, based on the rationale of providing a temporary three-dimensional (3D) structure for the growth of living cells and guide for tissue formation.^7^ Either osteochondral or chondral cell-free scaffolds reached the clinical practice, and studies are now being published with good mid- and long-term results.^8,9^

MaioRegen (Fin-Ceramica Faenza S.p.A., Italy) is a cell-free nano-structured, biomimetic implant for the treatment of chondral or osteochondral lesions with no or slightly/severely alteration of the subchondral bone. It is available in three different configurations (MaioRegen Prime, MaioRegen Slim, and MaioRegen Chondro+) sharing the same composition (type I collagen and magnesium-enriched hydroxyapatite) but with a different multilayer structure (bilayer or trilayer) tailored for a specific clinical indication.^10^ MaioRegen Prime, with a trilayer composite structure, is the most widely studied multilayered osteochondral scaffold to date. Currently available clinical evidence for the treatment of osteochondral knee defects with this scaffold reported promising satisfactory and reliable results at mid- and long-term follow-up, with a low rate of complications and failures.^10-13^

Differently from MaioRegen Prime, which mimics the entire osteochondral anatomy, MaioRegen Chondro+ is a bilayer scaffold consisting of a cartilage layer, composed of type I collagen, and a second layer reproducing calcified cartilage, composed of type I collagen and magnesium-enriched hydroxyapatite in a 60/40 ratio. Thus, it is indicated for the treatment of full-thickness chondral lesions with no or minor involvement of the subchondral bone (traumatic or posttraumatic grade-III lesions according to International Cartilage Regeneration & Joint Preservation Society (ICRS) Classification, grade III-IV Outerbridge lesions).

The purpose of this study was to assess the safety and clinical efficacy of MaioRegen Chondro+ in patients affected by articular knee lesions of different origin and localization. It was hypothesized that, based on equivalence composition with MaioRegen Prime, this new scaffold would be as safe as its predecessor; moreover, thanks to its bilayer composite structure, it is expected to be particularly effective in repairing moderately severe cartilage lesions that do not or only partially involve the subchondral bone.

## Methods

### Study Design and Patient Selection

This investigator-initiated prospective study was conducted on patients carrying symptomatic focal chondral lesions in the knee joint. Men and women aged >18 years with traumatic or degenerative focal cartilage defects of the knee and had failed conservative treatment were eligible for enrolment. Inclusion criteria for patients were symptomatic knee articular cartilage defects and verification by magnetic resonance imaging (MRI) examination. An MRI investigation of cartilage defects was performed for each patient before surgery, and an independent radiologist evaluated all MRI images. Lesions had to meet the following requirements: size >2 cm^2^ and grade >2 according to the ICRS classification; stable knee joints and normal leg axis were also required. Patients with local osteoarthritic changes >1° (Kellgren-Lawrence), valgus or varus deformities of the knee joint, or systemic diseases were excluded from the study. The following data were recorded for each patient: age and gender; number, location, size, IRCS grade and etiology of the lesion; associated pathologies; and treatment received. The study was approved by the Kaunas regional biomedical research committee (protocol number P1-BE-2-22; approval date: August 2, 2018); patients gave informed consent to participate in the study.

### Patient Population

Thirty-one patients meeting the inclusion criteria were enrolled in this study (4 females and 27 males). Demographics and lesion-related data are reported in **Table 1**. Mean age at the time of surgery was 38.9 years (range: 22-58). Most defects (88.0%) were graded 3/4 according to ICRS classification. A total of 19 patients (61.3%) presented associated pathologies such as meniscus or anterior cruciate ligament (ACL) lesions, or osteoarthritis. The lesions were of different origin, including traumatic (25.8%), posttraumatic (38.7%), and degenerative (35.5%). Most lesions (78.5%) were located at the femoral condyle (medial and lateral femoral condyle, 51.6% and 32.3%, respectively); 58.1% were located at the trochlea and 19.4% at the patella. The mean lesion size was 4.1 ± 1.68 (range: 1.0-6.5) in the MFC, 3.0 ± 1.80 (range: 1.5-6.0) in the LFC, 2.9 ± 1.16 (range: 1.5-4.0) in the patella, and 4.3 ± 1.29 (range: 2.0-6.0) in the trochlea.

**Table 1.** Patient Demographics and Lesion-Related Data.

Patients—N = 31	
Age	
Mean (range)—yr	38.9 (22-58)
Gender—no. (%)	
Female	4 (12.9)
Male	27 (87.1)
Knee—no. (%)	
Right	15 (48.4)
Left	16 (51.6)
Associated pathologies—no. (%)	
Lesion at lateral meniscus	8 (25.4)
Lesion at medial meniscus	3 (9.7)
Medial osteoarthritis	4 (12.9)
Treatment of anterior cruciate ligament	4 (12.9)
On the whole	19 (61.3)
Lesion etiology—no. (%)	
Degenerative	11 (35.5)
Posttraumatic	12 (38.7)
Traumatic	8 (25.8)
Number of treated sites per patient—no (%)	
1 site	21 (67.7%)
>1 site	10 (32.3%)
Lesion site—no. (%)	
MFC	16 (51.6)
LFC	10 (32.3)
Patella	6 (19.4)
Trochlea	18 (58.1)
Lesion size (cm^2^)—mean ± SD (range)	
MFC	4.1 ± 1.68 (1.0-6.5)
LFC	3.0 ± 1.80 (1.5-6.0)
Patella	2.9 ± 1.16 (1.5-4.0)
Trochlea	4.3 ± 1.29 (2.0-6.0)
ICRS grade 3-4, no (%)	
MFC	16 (51.6)
LFC	8 (25.8)
Patella	4 (12.9)
Trochlea	16 (51.6)
On the whole	44 (88.0)
Treatment—no. (%)	
No treatment	6 (12.0)
Chondro+	35 (70.0)
Microfractures	7 (14.0)
Debridement	2 (4.0)

ICRS = International Cartilage Regeneration & Joint Preservation Society ; MFC = Medial Femoral Condyle; LFC = Lateral Femoral Condyle.

Most patients (21, 67.7%) had a cartilage lesion at a single site; 10 patients (32.3%) had lesions at multiple sites. In total, 50 lesions were analyzed in this study: six were left untreated, 35 were treated by MaioRegen Chondro+ implantation, and nine by other procedures (seven by microfractures and two by debridement).

**Figure 1.**
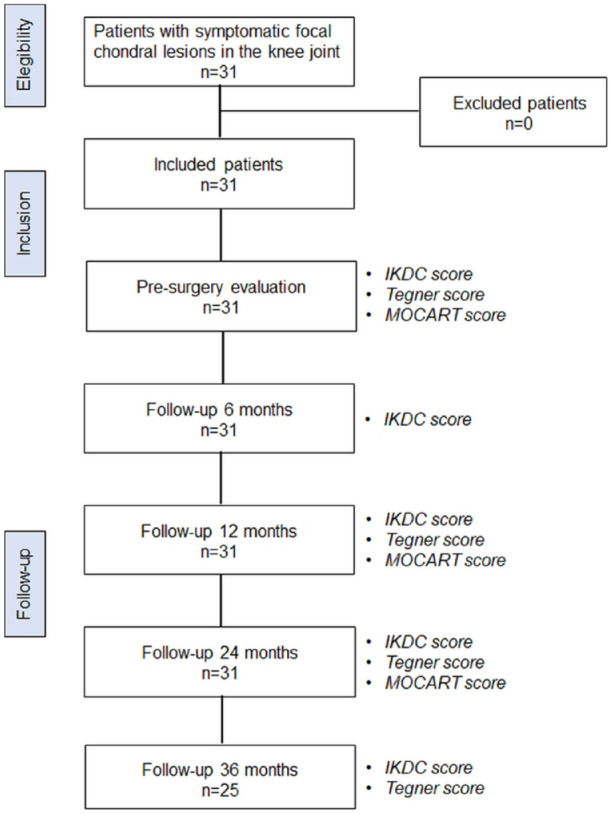
Flowchart of patient selection and follow-up. IKDC = International Knee Documentation Committee; MOCART, Magnetic Resonance Observation of Cartilage Repair Tissue.

All Chondro+-treated lesions were graded 3/4 according to ICRS classification; their mean size was 4.6 ± 1.13 cm^2^. ICRS grades, number, and mean size of lesions treated with MaioRegen Chondro+ implantation at different knee joint sites are reported in **Table 2**. All patients were followed up for 24 months. Of 31 initial patients, 25 were clinically evaluated 36 months after implantation. Demographics and lesion-related details of patients reaching the 36 months follow-up are reported in Suppl. Tables S1 and S2 in Supplementary Material. **Figure 1** details patient follow-up.

**Table 2.** Characteristics of Chondro+-Treated Lesions.

Lesions—N = 35		
Lesion site—no. (%)		
MFC	13	
LFC	5	
Patella	2	
Trochlea	15	
Lesion size (cm^2^)—mean ± SD (range)		
MFC	4.6 ± 1.29 (2.0-6.5)	
LFC	4.5 ± 1.27 (2.5-6.0)	
Patella	4.0 (4.0-4.0)	
Trochlea	4.6 ± 1.07 (3.0-6.0)	
All	4.6 ± 1.13 (2-6.5)	
ICRS grade 3/4—Subgroup: no. (%)		
	Grade 3	Grade 4
MFC	3c: 13 (100%)	—
LFC	—	4a: 5 (100%)
Patella	—	4a: 2 (100%)
Trochlea	3d: 1 (6.7%)	4a: 14 (93.3%)
All	3c: 13 (37.1%) 3d: 1 (2.9%)	4a: 21 (60%)

ICRS = International Cartilage Regeneration & Joint Preservation Society; MFC = Medial Femoral Condyle; LFC = Lateral Femoral Condyle.

Only in 17 (55%) of 31 cases Chondro+ implantation was performed as primary single procedure, while in 19 (61.3%) cases Chondro+ implantation was performed in association with other type of surgical interventions (nine medial meniscus, five lateral meniscus procedures, three cases with high tibial osteotomy, and two cases with ACL reconstruction) (**Table 3**). In three cases, the procedure was carried out after failure of other type of primary surgeries (one previously failed lateral meniscectomy, followed by additional Actifit meniscal scaffold implantation, one previously failed OAT, and one microfracture procedure).

**Table 3.** Additional Procedures Performed Concomitantly With Chondro+ Implantation.

Additional Procedures—N = 19	
Concomitant procedures—no. (%)	
Lateral meniscus suturing	3 (15.8%)
Lateral meniscus partial resection	2 (10.5%)
Medial meniscus suturing	2 (10.5%)
Medial meniscus partial resection	7 (36.8%)
ACL reconstructions	2 (10.5%)
HTO	3 (15.8%)

ACL = anterior cruciate ligament; HTO = high tibia osteotomy.

### Surgical Technique

MaioRegen Chondro+ was implanted using an arthroscopic approach, even if the device is usually implanted via a mini-arthrotomy technique. All surgical implantations were performed by a single experienced surgeon. *Arthroscopic Chondro+ implantation technique*.

**Figure 2.**
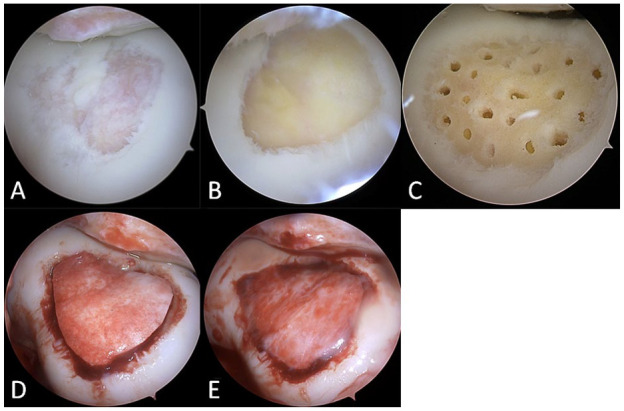
Chondro+ scaffold implantation in a trochlea chondral lesion. (**A**) Grade-IV articular cartilage defect; (**B**) implant site preparation by cleaning and removal of the calcified layer; (**C**) microfractures before Chondro+ implantation; (**D**) Chondro+ implantation; and (**E**) graft fixation with fibrin glue.

During the arthroscopic Chondro+ implantation procedure, the loose chondral flaps and degenerated cartilage were debrided down to the subchondral bone using shaver blade and small burr until the viable subchondral bone and stable cartilaginous rims were reached. The area of the articular cartilage defect was estimated and measured with an arthroscopic hook probe, and the final defect size was calculated in square centimeters. All remaining free cartilage debris were removed with shaver suction and, as recommended in the surgical technique of the osteochondral scaffold, the subchondral bone penetration procedure was performed in all patients using a 1.5-mm Kirschner wire or PowerPick™ Microfracture Instrument (Arthrex GmbH, Munich, Germany). Then, after the water was removed from the joint with shaver suction, the scaffold was cut to perfectly fit the size of the defect site and was soaked in sterile water for 1 to 2 minutes. The scaffold was hence inserted through the knee joint and gently press-fitted into the defect through a 5-ml syringe tube with an arthroscopic hook in dry arthroscopy conditions. When well-adjusted, the scaffold was fixed in place using fibrin glue (Tisseel, Baxter, Westlake Village, California, USA). After the fixation, mechanical stability of the implant was checked in dry arthroscopy conditions by quick knee flexion and extension movements (**Fig. 2**). Extra fibrin clots were removed from the knee joint after the scaffold fixation with a shaver suction. No scaffold displacement was recorded after the fixation, and in all cases, the scaffold was stable and maintained in place after several knee hyperflexion and hyperextension movements.

### Additional Procedures

All concomitant injuries (19; 61.3%) were treated at the same time with Chondro+ scaffold implantation procedures. Type and number of additional procedures performed concomitantly with Chondro+ implantation is presented in **Table 3**.

### Postoperative Recovery

The first day after the surgery, an extra blood evacuation from the knee joint was performed for 11 (35%) patients. Regardless, cartilage lesion treated, all the patients followed the same postoperative rehabilitation protocol, consisting of partial weight bearing for 2 to 4 weeks followed by full weightbearing in 4–6 weeks. The first day postsurgery, a continuous passive motion (CPM) machine was used for every patient, starting from 45° of flexion and full extension adding 10° each day. The CPM machine was used until full range of knee motion was achieved passively. Then, in 4 to 6 weeks accordingly to the main and concomitant surgeries performed, patients were allowed to start full weightbearing according to the symptoms. Sport activities were allowed approximately 6 to 8 months after surgery.

### Outcome Measures

For clinical evaluation, the International Knee Documentation Committee (IKDC) score^14^ and the Tegner activity scale^15^ were used to determine joint function and return to preinjury physical activities. IKDC measurements were performed preoperatively (baseline) and at 6, 12, and 24 months after treatment. Tegner activity score was assessed before injury, preoperatively, and at 12 and 24 months after surgery. All follow-up examinations were performed by independent investigator.

All 1.5 or 3 Tesla MRI investigations were performed on MAGNETOM Altea syngo MR XA11 system using a dedicated knee coil at an average of 11.6 (7-14) months after surgery. The MRI protocol was identical for all MRI examinations and consisted of the following sequences: sagittal proton density-weighted (PDW) fat-saturated (FS), sagittal T-2 weighted (T2w) turbo-spin-echo (TSE), sagittal PD-TSE-FS, sagittal T2-TSE, and coronal PD-TSE-FS. The MOCART (Magnetic Resonance Observation of Cartilage Repair Tissue) 2.0 knee scoring system was applied for the assessment of cartilage repair. All imaging evaluations were performed by an independent radiologist.

### Statistical Analysis

Descriptive statistics are provided for all variables in the summary tables. Quantitative variables are summarized on nonmissing observations by using n, mean, standard deviation (SD), median and range (minimum and maximum). Categorical variables are presented using absolute frequencies and percentages. For quantitative efficacy variables, 95% confidence interval (CI) of the mean is presented. Student’s t test was applied on the data for MOCART, IKDC, and Tegner scores to test change from baseline at visits of follow-up. Statistical analysis was performed using SAS (Statistical Analysis System) Software (Cary, North Carolina, USA).

## Results

### Clinical Results

**Figure 3.**
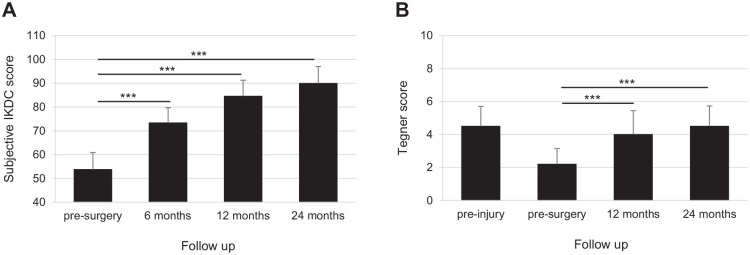
Clinical efficacy evaluation. (**A**) Mean value of the subjective IKDC scores collected presurgery (baseline) and at 6, 12, and 24 months follow-up. (**B**) Mean value of the Tegner activity score collected preinjury, presurgery (baseline) and at 12, and 24 months follow-up. The *P* values indicate the difference between presurgery and follow-up values. IKDC = International Knee Documentation Committee. ***P < 0.001.

The clinical efficacy of the treatment was investigated by the IKDC subjective score and the Tegner activity score. Both scores improved significantly from preoperative level to 24-month follow-up. IKDC mean changes were statistically significant at all time points, increasing from 53.7 ± 7.17 (baseline score) to 73.3 ± 6.41 after 6 months, with an improvement of 19.5 ± 7.27 (95% CI: 16.9-22.2, *P* < 0.001), and to 84.5 ± 6.78 after 12 months with a change from presurgery of 30.8 ± 7.63 (95% CI: 28.0-33.6, *P* < 0.001); at 24 months follow-up, the IKDC subjective score reached a value of 89.9 ± 7.15 with an improvement of 36.2 ± 8.00 points from the baseline value (95% CI: 33.3-39.2, *P* < 0.001) (**Fig. 3A**). The Tegner score improved significantly from preoperative assessment (2.2 ± 0.95) to 4.0 ± 1.45 (change of −0.6 ± 0.62; 95% CI: −0.8 to −0.4, *P* < 0.001) and 4.5 ± 1.23 (improvement of −0.0 ± 0.18; 95% CI: −0.1 to 0.0, *P* < 0.001) at the 1-year and 2-year postoperative follow-up, respectively, and reached the preinjured activity level (4.5 ± 1.21) (**Fig. 3B**). Notably, both scores improvement was maintained over time in those patients who reached the 3-year follow-up (Suppl. Fig. S1 in Supplementary Material).

**Figure 4.**
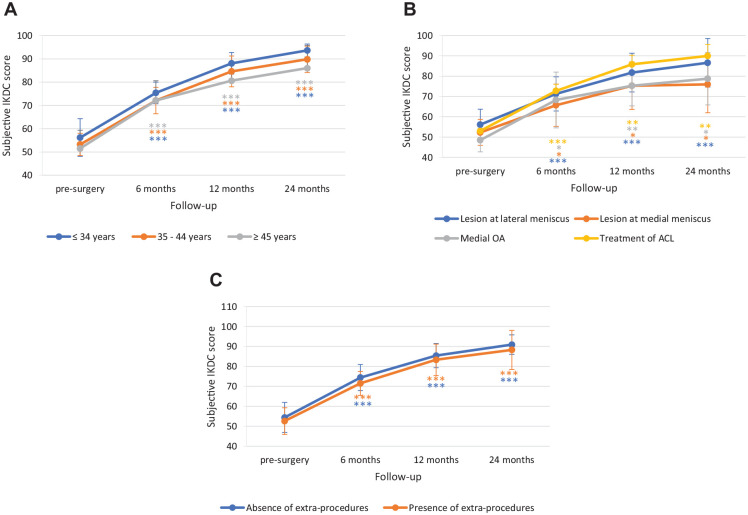
Subgroup analysis for IKDC score by (**A**) age, (**B**) associated diseases, and (**C**) absence/presence of extra-procedures. The *P* values indicate the difference between presurgery and follow-up values. IKDC = International Knee Documentation Committee; ACL = anterior cruciate ligament; OA = osteoarthritis. *P < 0.05, **P < 0.01, ***P < 0.0001.

**Figure 5.**
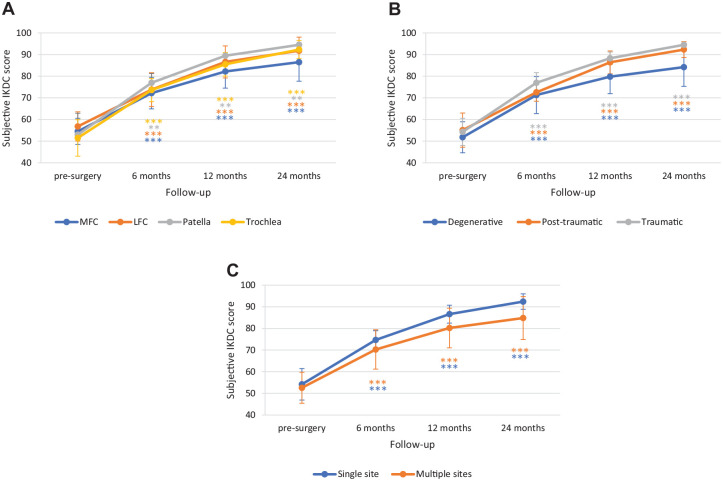
Subgroup analysis for IKDC score by (**A**) lesion site, (**B**) etiology, and (**C**) number of treated sites. The *P* values indicate the difference between presurgery and follow-up values. IKDC = International Knee Documentation Committee. **P < 0.01, ***P < 0.0001.

To identify any subgroups of patients who may, or may not, benefit of the treatment, the efficacy of Chondro+ implantation was further evaluated by performing subgroup analyses based on preintervention clinical demographic data (age, associated diseases, and absence/presence of extra-procedures) (**Fig. 4**) and on lesion characteristics (site, number, and etiology of the lesions) (**Fig. 5**).

No differences were observed in IKDC score improvement according to age (improvement at 24 months: ≤34 years, 37.5 ± 7.79; 35-44 years, 36.6 ± 6.88; ≥45 years, 34.5 ± 9.65), associated disease (lesion at lateral meniscus 30.5 ± 7.95; lesion at medial meniscus 23.7 ± 7.77; medial OA 30.3 ± 12.69; treatment of ACL 37.0 ± 7.44) or absence/presence of extra-procedures (absence 36.5 ± 6.4; presence 35.8 ± 10.35). As shown in **Figure 5A**, the improvement in IKDC score was similar at the different knee joint sites: from baseline to 24-months follow-up, it increased by 32.1 ± 8.07 points at MFC, 35.0 ± 4.95 points at LFC, 41.5 ± 9.19 points at patella, and 40.7 ± 6.68 points at trochlea. Similarly, no statistically significant differences emerged from the subgroup analysis considering the etiology of the lesions (**Fig. 5B**). The improvement in IKDC score from baseline to 24 months follow-up was similar for lesions of degenerative (32.4 ± 9.41 points), traumatic (40.1 ± 5.79 points), and posttraumatic (37.2 ± 6.77 points) origin. The presence of single or multiple lesions also did not affect treatment efficacy (**Fig. 5C**), as it was associated with a comparable increase in IKDC scores from baseline to 24 months follow-up: single site = 38.1 ± 7.66 points; multiple sites = 32.2 ± 7.51 points.

**Figure 6.**
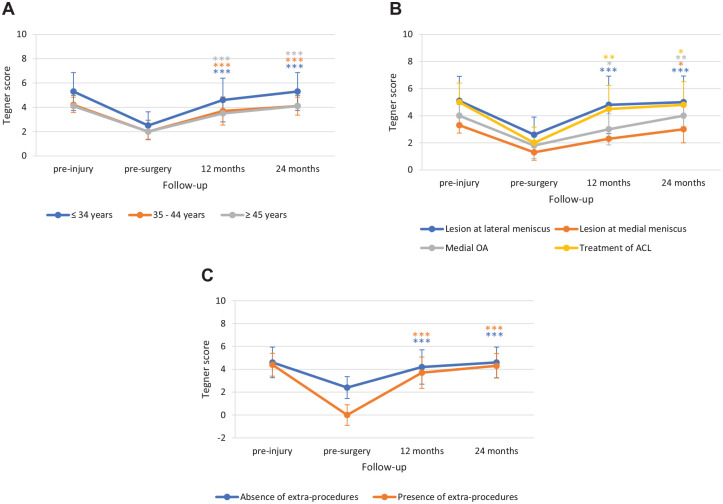
Subgroup analysis for Tegner score by (**A**) age, (**B**) associated diseases, and (**C**) absence/presence of extra-procedures. The *P* values indicate the difference between presurgery and follow-up values. ACL = anterior cruciate ligament; OA = osteoarthritis. *P < 0.05, **P < 0.01, ***P < 0.001.

**Figure 7.**
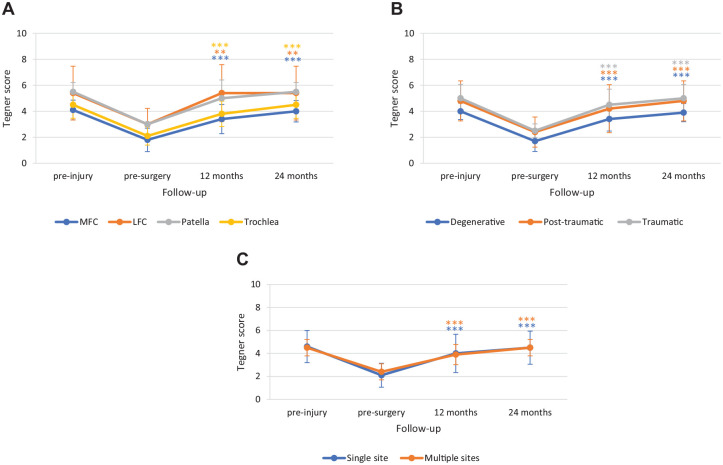
Subgroup analysis for Tegner score by (**A**) lesion site, (**B**) etiology, and (**C**) number of treated sites. The *P* values indicate the difference between presurgery and follow-up values. MFC = Medial Femoral Condyle; LFC = Lateral Femoral Condyle. **P < 0.01, ***P < 0.001.

Likewise, no differences were observed in Tegner score improvement according to age (improvement at 24 months: ≤34 years, 2.7 ± 0.79; 35-44 years, 2.1 ± 0.57; ≥45 years, 2.1 ± 0.32), associated disease (lesion at lateral meniscus 2.4 ± 0.74; lesion at medial meniscus 1.7 ± 0.6; medial OA 2.3 ± 0.5; treatment of ACL 2.8 ± 0.96), or absence/presence of extra-procedures (absence 2.3 ± 0.65; presence 2.4 ± 0.67) (**Fig. 6**). Similar improvements were also observed according to lesion sites (MFC 2.2 ± 0.55; LFC 2.4 ± 0.89; patella 2.5 ± 0.71; trochlea 2.5 ± 0.69), lesion etiology (degenerative 2.2 ± 0.54; posttraumatic 2.3 ± 0.78; traumatic 2.5 ± 0.76) or number of treated sites (single sites 2.4 ± 0.75; multiple sites 2.1 ± 0.32). In all cases, the preinjury Tegner level was reached (**Fig. 7**).

Moreover, IKDC and Tegner scores in patients who reached the 3-year follow-up displayed no significant differences up to 36 months regardless lesion sites, etiology, or number of treated sites (Suppl. Figs. S2 and S3 in Supplementary Material).

### MRI Results

At 12 months, the mean MOCART score significantly increased from 31.9 ± 8.23 at baseline to 68.2 ± 10.61, with an improvement of 36.3 ± 7.18 points (95% CI: 33.7-38.9, *P* < 0.001) (**Table 4**). Also, all MOCART 2.0 subscales displayed improvements at 1 year (volume of cartilage defect filling 10.2 ± 3.29 [95% CI: 5-15, *P* < 0.001]; integration into adjacent cartilage 8.5 ± 3.70 [95% CI: 0-15, *P* < 0.001]; surface of the repair tissue 3.1 ± 3.34 [95% CI: 1.8-4.3, *P* < 0.001]; structure of the repair tissue 3.5 ± 4.86 [95% CI: 0-10, *P* < 0.001]; signal intensity of the repair tissue 8.9 ± 4.22 [95% CI: 0-15, *P* < 0.001]; bony defect or bony overgrowth −1.0 ± 2.01 [95% CI: −5 to 0, *P* = 0.012]; subchondral changes 3.7 ± 3.64 [95% CI: −5 to 10, *P* < 0.001]; data not shown).

**Table 4.** Results of MOCART Score.

	Follow-up		
	Presurgery	12 Months	Change From Baseline
MOCART			
All patients (N = 31)	31.9 ± 8.23	68.2 ± 10.61	36.3 ± 7.18***
MOCART according to lesion site—mean ± SD			
MFC	30.0 ± 8.90	65.4 ± 10.89	35.4 ± 9.00***
LFC	31.0 ± 8.22	67.0 ± 12.04	36.0 ± 4.18***
Patella	32.5 ± 3.54	67.5 ± 10.61	35.0 ± 7.07
Trochlea	34.5 ± 8.20	72.3 ± 9.84	37.7 ± 6.47***
MOCART according to etiology—mean ± SD			
Degenerative	28.8 ± 10.07	65.5 ± 13.50	37.3 ± 7.20***
Posttraumatic	32.9 ± 6.56	66.3 ± 9.08	33.3 ± 8.35***
Traumatic	35.6 ± 6.23	75.0 ± 4.63	39.4 ± 3.20***
MOCART according to no. of treated sites—mean ± SD			
Single site	32.4 ± 8.16	68.3 ± 9.26	36.0 ± 6.45***
Multiple sites	31.0 ± 8.76	68.0 ± 13.58	37.0 ± 8.88***

MOCART = Magnetic Resonance Observation of Cartilage Repair Tissue; MFC = Medial Femoral Condyle; LFC = Lateral Femoral Condyle.

*** P < 0.001.

No differences were observed in MOCART score improvement according to lesion sites (MFC 35.4 ± 9.00; 36.0 ± 4.18; patella 35.0 ± 7.0; trochlea 37.7 ± 6.47), lesion etiology (degenerative 37.3 ± 7.2; posttraumatic 33.3 ± 8.35; traumatic 39.4 ± 3.2) or number of treated sites (single sites 36.0 ± 6.45; multiple sites 37.0 ± 8.88). Similar results were observed in patients reaching the 36 months follow-up (Suppl. Table S3 in Supplementary Material).

**Figure 8.**
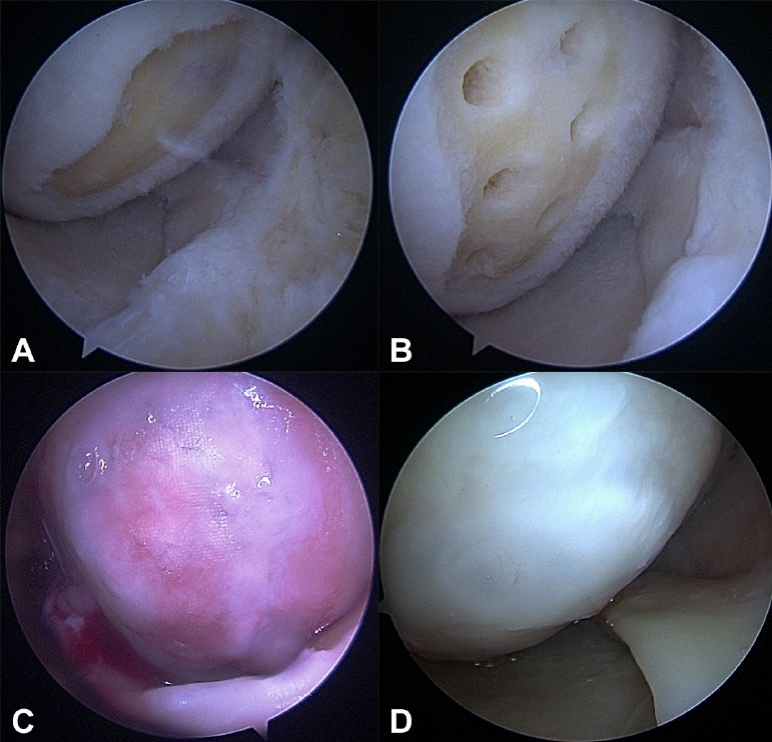
Sample case: a 59-year-old patient with grade-I osteoarthritis and grade-IV chondral lesion at the medial femoral condyle. (**A**) Presurgery; (**B**) after microfracture; (**C**) after Chondro+ scaffold implantation into Medial Femoral Condyle (MFC) and fibrin glue fixation; (**D**) second-look arthroscopy at 36 months follow-up. The procedure was performed at the time of high tibia osteotomy (HTO) plate removal, not due to Chondro+-related adverse events.

In **Figure 8**, an example of lesion recovery after Chondro+ scaffold implantation is reported, showing good integration of the device and complete filling of the defect with new articular cartilage tissue.

**Figure 9.**
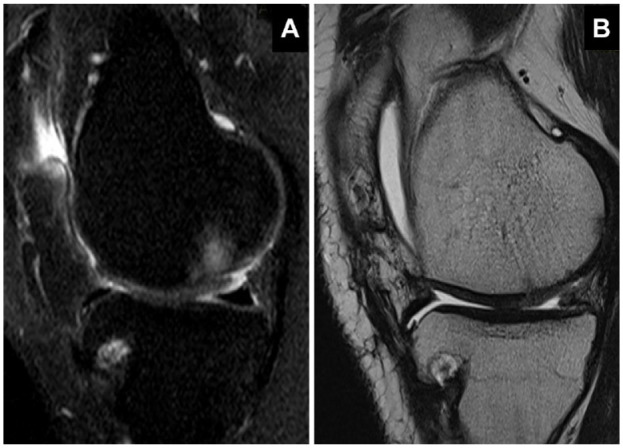
MRI imaging of a Chondro+-treated subject knee before scaffold implantation (**A**) and at 7 months of follow-up (**B**). (**A**) Sagittal proton density-weighted fat-saturated (PDW-FS) image showing the focal chondral defect of the Medial Femoral Condyle (MFC) with thinning of the cartilage. (**B**) Sagittal T2-TSE (turbo-spin-echo) image showing the regenerated cartilage tissue with focal hyperintense area at 7 months after Chondro+ implantation. MRI = magnetic resonance imaging.

**Figure 9** illustrates representative MRI imaging of a Chondro+-treated subject knee before scaffold implantation (A) and at 7 months of follow-up (B).

No after-surgery infections, synovitis, or any adverse events were reported in the entire population analyzed. Also, none of the patients were reoperated because of the failure of the articular cartilage procedure with Chondro+ implantation in this investigation period.

## Discussion

The main finding of this study was that the implantation of a cell-free osteochondral scaffold provided significant clinical benefits in the treatment of ICRS grade 3 to 4 knee joint lesions at a 2-year follow-up. The clinical improvement was observed regardless of the etiology of the lesion, its location, and the presence of single or multiple defects in the knee joint. Furthermore, these results were maintained in a subset of patients who reached a 3-year follow-up.

The technique of scaffold implantation has gained more and more interest in recent years; thanks to their ability to promote tissue repair by providing both structural and functional cues to cells, scaffolds represent an appealing single-step and morbidity-free procedure for the management of cartilage lesions.

Many studies have shown the safety and efficacy of a 3D acellular scaffold composed of three layers of equine type I collagen and magnesium-enriched hydroxyapatite nanocrystals (MaioRegen Prime). This scaffold mimics the entire bone/cartilage system and is specifically designed to treat knee cartilage defects with severe subchondral bone involvement. It provided significant^16-19^ and long-term (up to 10 years follow-up)^8,13^ clinical improvement in the treatment of knee osteochondral lesions; it also showed satisfactory clinical outcomes in patients affected by osteochondritis dissecans^19,20^ and osteoarthritis.^21,22^

Surgical treatment of focal knee cartilage defects is indicated in the presence of symptomatic defects, with the short-term goal of improving pain and function and the long-term hope of avoiding or at least delaying the progression to further degeneration and osteoarthritis.^23,24^ Deep cartilage lesions (IRCS grade 3-4) may cause pain and functional impairment even in the absence of substantial subchondral bone involvement; also in this case, given the poor regenerative capacity of the cartilaginous tissue, the indication is surgery. In this study, the safety and efficacy of a new 3D cell-free scaffold (MaioRegen Chondro+), with a bilayer composite structure designed to treat full-thickness chondral lesions with no or minor involvement of the subchondral bone, have been investigated.

The treated lesions were deep (mostly grade 3-4 according to IRCS classification) and quite large (size range: 1.5-6.5 cm^2^). A statistically significant improvement in IKDC subjective score compared with the basal evaluation was observed at 6, 12, and 24 months after scaffold implantation in all treated patients (n = 31). Interestingly, subgroup analysis by etiology, location, and number of treated defects evidenced no differences in the treatment outcome. These results support a comparable efficacy for the biphasic scaffold following single or multiple implantations at different knee joint sites (nonsignificant best-to-worse trend: patella, trochlea, LFC, and MFC); efficacy also appears to be independent of the nature of the lesion (traumatic, posttraumatic, or degenerative).

Notably, the Tegner scores documented a substantial clinical improvement as early as 12 months after surgery, showing similar score with respect of preinjury values. Again, similar results were obtained regardless the lesion site, the lesion etiology, or the number of treated sites. Overall, the clinical results were similar to those reported by MaioRegen Prime.^10^

It should be underlined that in this study population showed higher IKDC values than expected: this could be an inherent factor in the type of injuries that are the target of the treatment because in the absence of severe subchondral bone involvement, symptoms might be less severe. In addition, the mean age is 40 years: considering that the IKDC score is largely based on sport-like activities, an age-related decrease in the performance of hard exercise could result in lower values in a middle-aged population than in a younger one and negatively affect the outcome scores. The two factors might contribute to underestimate the gap between pretreatment and posttreatment IKDC scores. All patients were included in the safety analysis. No adverse events were recorded, thus confirming the safety profile of the previously developed triphasic scaffold.

The MOCART 2.0 knee score, an incremental update on the original MOCART score,^25^ was used for a longitudinal evaluation of the recovery status of chondral lesions. The values increased from preoperatively up to 1 year postoperatively, indicating an improvement of the MRI aspect of the repair tissue. The analyses performed on a subset of treated patients who reached the 3-year follow-up (N = 25) further corroborated the efficacy and safety of the scaffold. Despite the low number of subjects with 36-month follow-up, these results are interesting because, considering the variables that might influence the performance outcome, the subgroup of patients at 36 months did not differ from the overall sample. Therefore, we could infer that the results for this subset is not different from what we would expect for the total subjects.

Limitation of this study include the lack of a control or comparison group and the relatively small number of patients enrolled, which nevertheless was sufficient to detect an improvement over time. The heterogeneity of the patients, in terms of etiology, lesion size and localization, presence of associated pathologies, with several of them needing extra-procedures, might also be a limitation; however, this better reflect the clinical scenario allowing for a more representative sample of the general population requiring cartilage lesion treatments. Studies on a larger cohort of patients and longer follow-up would be needed to confirm the efficacy and duration of the treatment. Despite the focus of this study laid on the clinical and imaging evaluation, another limitation is the absence of histological assessment to better understand the quality of the scaffold-induced regenerated tissues. However, routine biopsies in patients otherwise not requiring surgery were not performed due to ethical concerns.

Notwithstanding these limitations, this is the first study highlighting the safety and potential of a MaioRegen Chondro+ for the treatment of chondral lesions in the knee joint. The peculiar bilayer structure makes this scaffold particularly suitable for the treatment of cartilage lesions without subchondral bone involvement. Indeed, it allows the underlying bone structure to be preserved while promoting scaffold integration, thanks to the tidemark-like layer resembling calcified cartilage. Clinically, Chondro+ provided a significant improvement in IKDC and Tegner activity scores at 2 years postoperatively, which was maintained over time in those patients who reached the 3-year follow-up. In addition, MRI evaluation at 12 months showed significant recovery of chondral lesions. This biphasic scaffold seems to be a safe and effective option for the treatment of moderately severe knee cartilage lesions that do not or only partially involve the subchondral bone.

## Supplemental Material

sj-docx-1-car-10.1177_19476035241232061 – Supplemental material for Arthroscopic Implantation of a Cell-Free Bilayer Scaffold for the Treatment of Knee Chondral Lesions: A 2-Year Prospective Study Supplemental material, sj-docx-1-car-10.1177_19476035241232061 for Arthroscopic Implantation of a Cell-Free Bilayer Scaffold for the Treatment of Knee Chondral Lesions: A 2-Year Prospective Study by Rimtautas Gudas, Mantas Staškūnas, Justinas Mačiulaitis, Emilė Gudaitė and Ieva Aleknaite-Dambrauskiene in CARTILAGE
