# Supplementary material for: Arthroscopic Implantation of a Cell-Free Bilayer Scaffold for the Treatment of Knee Chondral Lesions: A 2-Year Prospective Study
Source: Cartilage. 2024 Mar 19;16(1):5–16. doi: 10.1177/19476035241232061 (PMC11569651; doi:10.1177/19476035241232061)
Supplement: sj-docx-1-car-10.1177_19476035241232061 – Supplemental material for Arthroscopic Implantation of a Cell-Free Bilayer Scaffold for the Treatment of Knee Chondral Lesions: A 2-Year Prospective Study [file sj-docx-1-car-10.1177_19476035241232061.docx]

**Supplementary data**

Figure S1. Clinical efficacy evaluation in patients reaching the 36-month follow-up. A. Mean value of the subjective IKDC scores collected pre-surgery (baseline) and at 6, 12, 24, and 36 months follow up. ****, p<0.0001. B. Mean value of the Tegner activity score collected preinjury, pre-surgery (baseline) and at 12, 24, and 36 months follow up. ****, p<0.0001


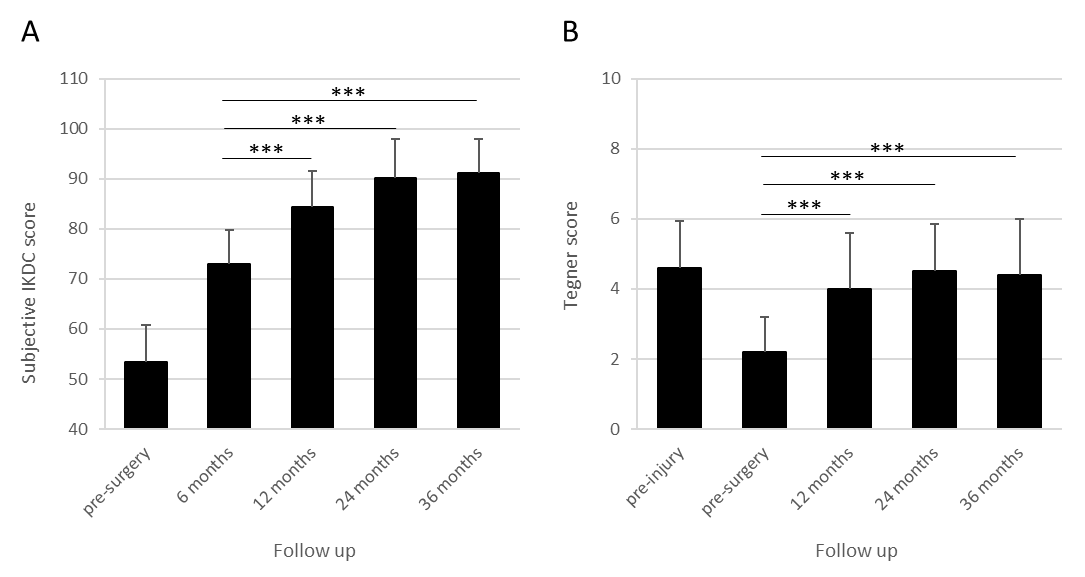


Figure S2. Subgroup analysis for IKDC score in patients reaching the 36-month follow-up by (A) age, (B) associated diseases, and (C) absence/presence of extra-procedures. *, p<0.05; **, p<0.01; ***, p<0.0001. The p values indicate the difference between baseline and follow-up values.


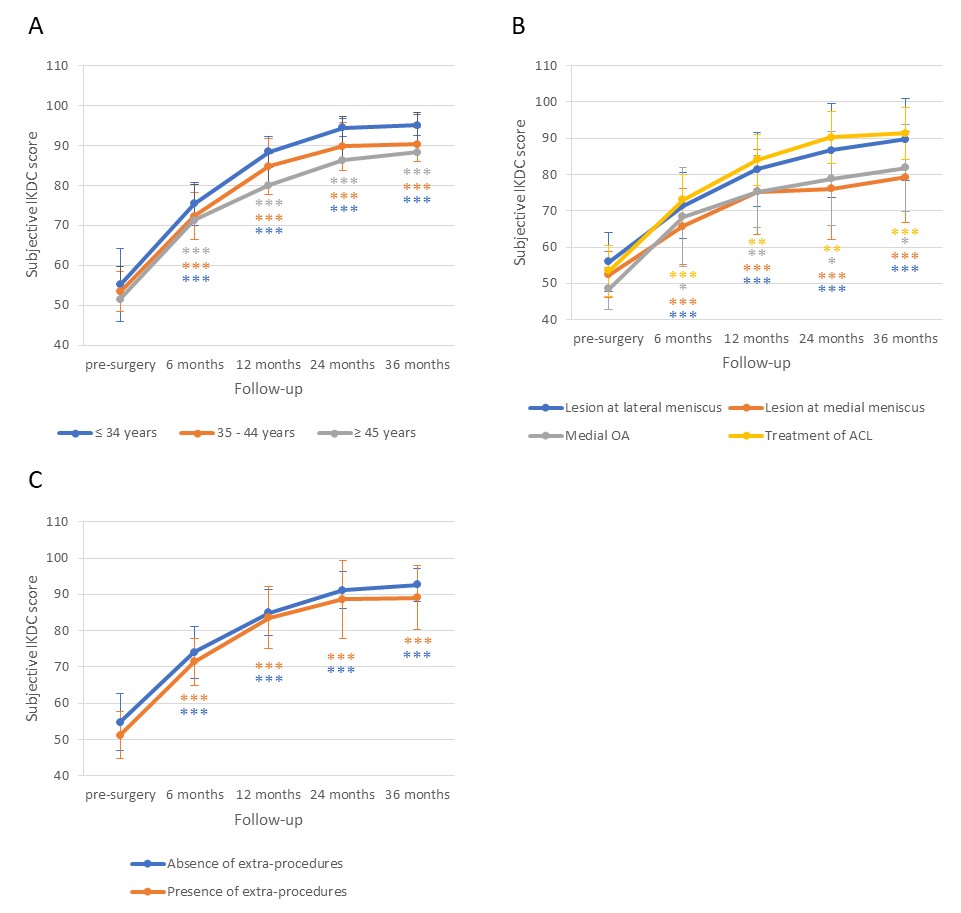


Figure S3. Subgroup analysis for IKDC score in patients reaching the 36-month follow-up by (A) lesion site, (B) etiology, and (C) number of treated sites. **, p<0.01; ***, p<0.0001. The p values indicate the difference between baseline and follow-up values.


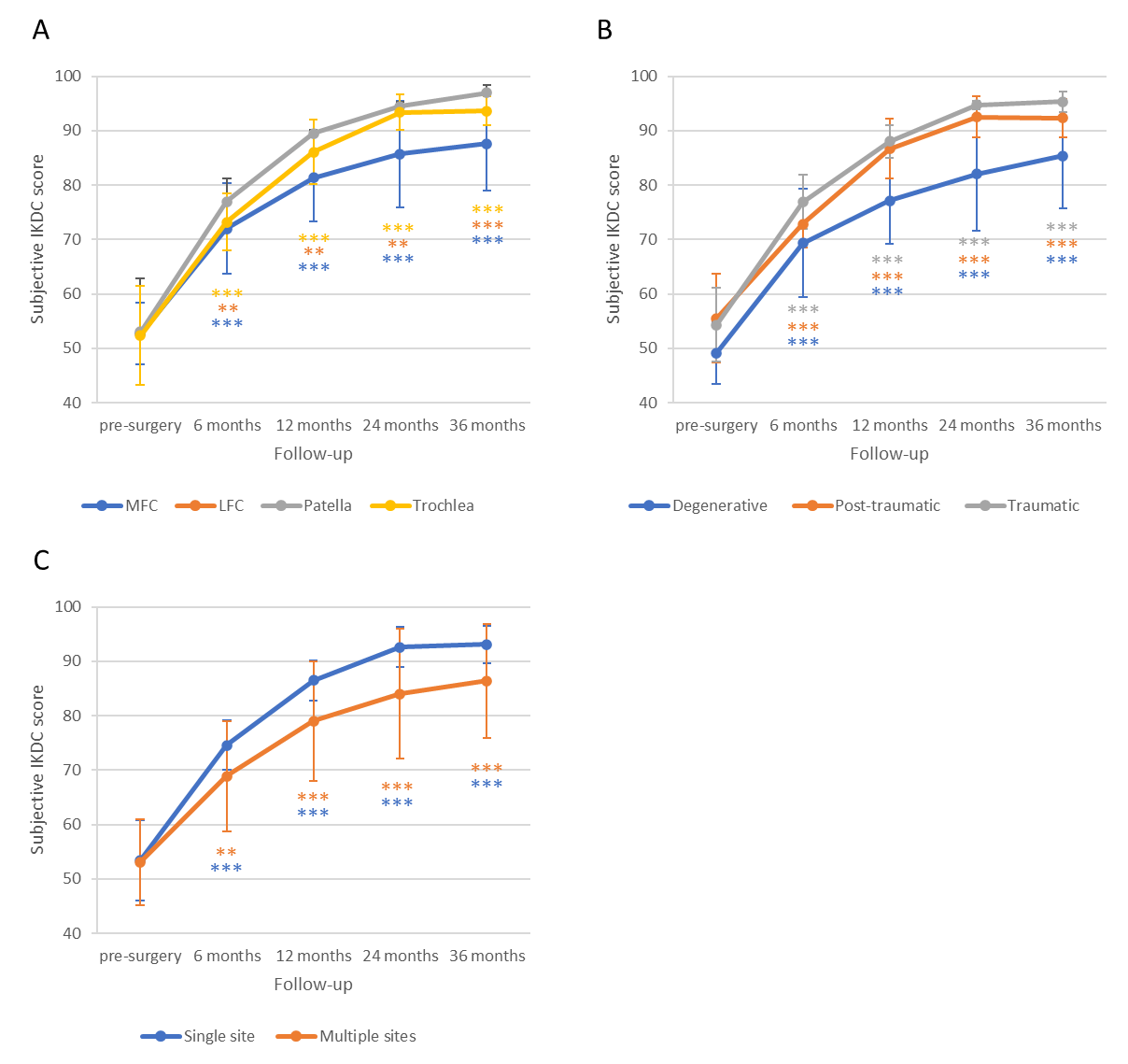


Figure S4. Subgroup analysis for Tegner score in patients reaching the 36-month follow-up by by (A) age, (B) associated diseases, and (C) absence/presence of extra-procedures. *, p<0.05; **, p<0.01; ***, p<0.0001. The p values indicate the difference between baseline and follow-up values.


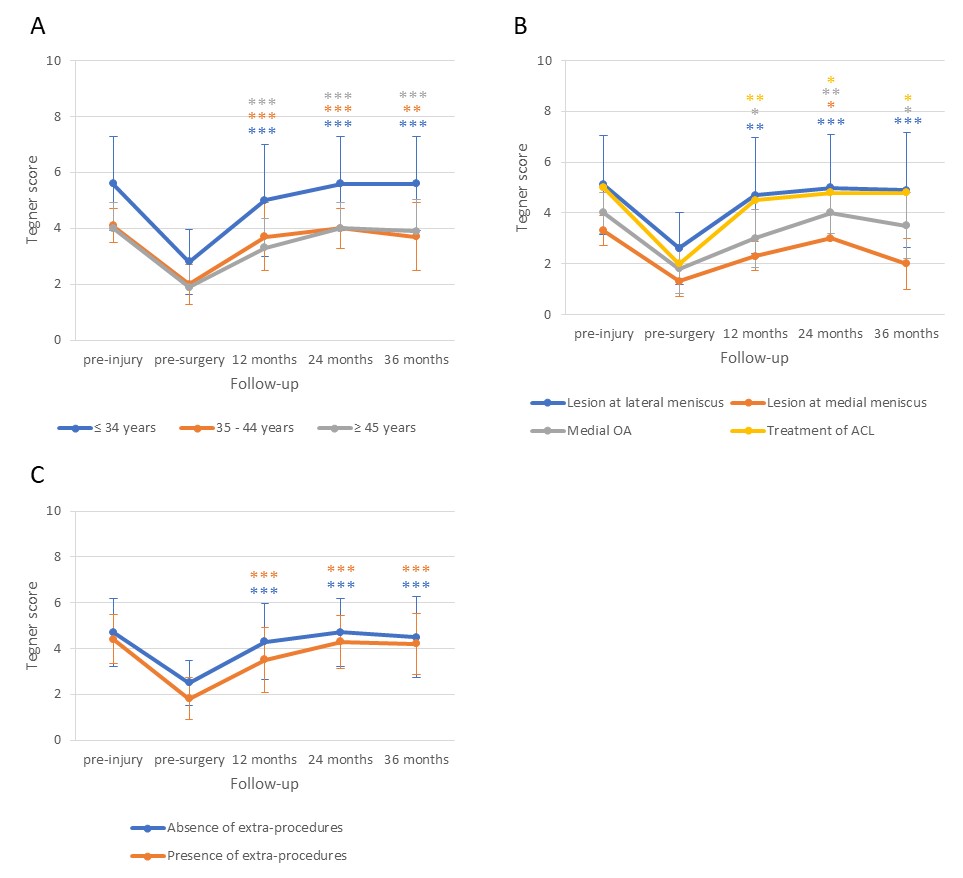


Figure S5. Subgroup analysis for Tegner score in patients reaching the 36-month follow-up by (A) lesion site, (B) etiology, and (C) number of treated sites. *, p<0.05; **, p<0.01; ***, p<0.0001. The p values indicate the difference between baseline and follow-up values.


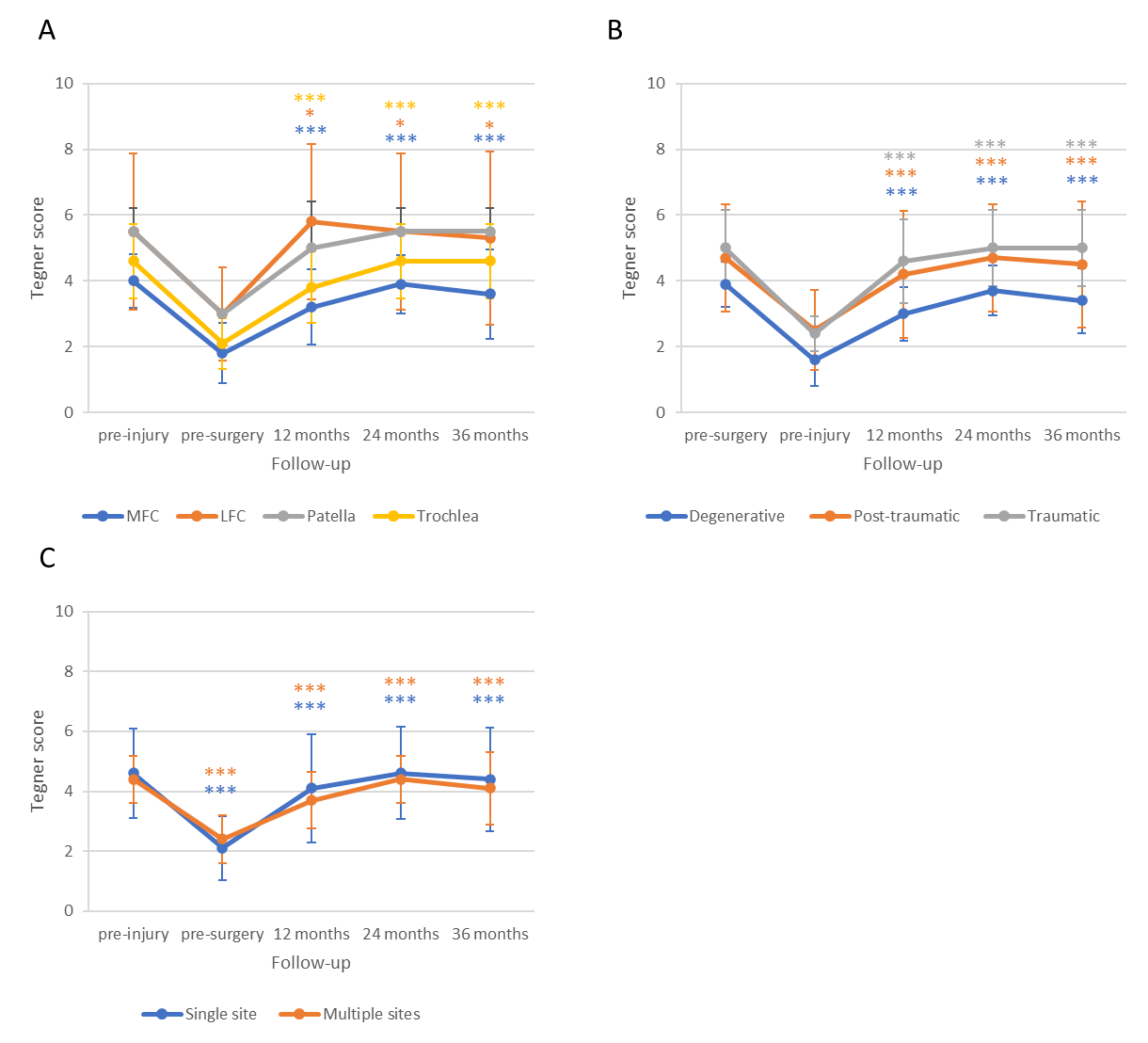


Table S1. Patient demographics and lesion-related data in patients reaching the 36-month follow-up.

Table S2. Characteristics of Chondro Plus treated lesions in patients reaching the 36-month follow-up.

| **Lesions - N = 28** |  |  |
| --- | --- | --- |
| *Lesion site* - no. (%) |  |  |
| MFC | 10 |  |
| LFC | 4 |  |
| Patella | 2 |  |
| Trochlea | 12 |  |
| *Lesion size (cm^2^)* - mean ± SD (range) | |  |
| MFC | 4.8 ± 1.08 (3.0-6.5) |  |
| LFC | 4.5 ± 1.47 (2.5-6.0) |  |
| Patella | 4.0 (4.0-4.0) |  |
| Trochlea | 4.8 ± 1.08 (3.0-6.0) |  |
| *ICRS grade 3-4* |  |  |
|  | *Grade 3* | *Grade 4* |
| MFC | 3c: 10 (100.0%) | - |
| LFC | - | 4a: 4 (100.0%) |
| Patella | - | 4a: 2 (100.0%) |
| Trochlea | - | 4a: 12 (100.0%) |
| All | 3c: 10 (35.7%) | 4a: 18 (64.3%) |

Table S3. Results of MOCART score in patients reaching the 36-month follow-up. **, p<0.01; ***, p<0.001

|  |  | Follow-up |  |
| --- | --- | --- | --- |
|  | Pre-surgery | 12 months | Change from baseline |
| *MOCART* |  |  |  |
| Patients at 36 months follow-up (N = 25) | 30.6 ± 7.54 | 67.0 ± 9.79 | 36.4 ± 7.29*** |
| *MOCART according to lesion site* - mean ± SD | |  |  |
| MFC | 28.5 ± 7.47 | 64.0 ± 10.49 | 35.5 ± 10.12*** |
| LFC | 28.8 ± 7.50 | 63.8 ± 11.09 | 35.0 ± 4.08*** |
| Patella | 32.5 ± 3.54 | 67.5 ± 10.61 | 35.0 ± 7.07 |
| Trochlea | 33.3 ± 8.29 | 71.7 ± 7.91 | 38.3 ± 5.00*** |
| *MOCART according to etiology* - mean ± SD | |  |  |
| Degenerative | 22.9 ± 2.67 | 60.0 ± 10.00 | 37.1 ± 8.09*** |
| Post-traumatic | 32.7 ± 6.84 | 66.8 ± 9.29 | 34.1 ± 8.31*** |
| Traumatic | 35.0 ± 6.45 | 74.3 ± 4.50 | 39.3 ± 3.45*** |
| *MOCART according to n. of treated sites* - mean ± SD | |  |  |
| Single site | 31.4 ± 7.24 | 66.9 ± 8.60 | 35.6 ± 6.84*** |
| Multiple sites | 28.6 ± 8.52 | 67.1 ± 13.18 | 38.6 ± 8.52*** |
